# Male Gonads Transplantation from Kadaknath Chicken to Chicken and Duck Surrogates

**DOI:** 10.3390/genes14051094

**Published:** 2023-05-16

**Authors:** Adnan Naim, Surya Kanta Mishra, Anjan Sahoo, Indra mani Nath

**Affiliations:** 1 KIIT-TBI, Campus 11, KIIT University, Bhubaneswar 751024, Odisha, India; 2 Indian Council of Agriculture and Research—Directorate of Poultry Research, Regional Centre, Bhubaneswar 751003, Odisha, India; 3 College of Veterinary Science and Animal Husbandry, Orissa University of Agriculture and Technology, Bhubaneswar 751003, Odisha, India

**Keywords:** Kadaknath, gonad, transplantation, interspecies, poultry

## Abstract

Transplantation of the gonadal tissue of male and female avian species, such as chicken, onto suitable surrogates and production of live offspring has been successfully demonstrated as a strategy for the conservation and re-constitution of valuable chicken germplasm. The main objective of this study was to establish and develop the male gonadal tissue transplantation technology for the conservation of the indigenous chicken germplasm. The male gonads of the Indian native chicken breed, Kadaknath (KN), were transplanted from a day-old donor to a recipient white leghorn (WL) chicken, and Khaki Campbell (KC) ducks, as surrogates. All the surgical interventions were performed under permitted general anaesthesia, and the chicks, upon recovery, were reared with and without immunosuppressant. The recipient surrogates for the donor KN gonads were housed and reared for 10–14 weeks, and post-sacrifice, developed gonadal tissues were harvested to squeeze out the fluid to perform artificial insemination (AI). The AI-entailed fertility test using the recovered seminal extract from the transplanted KN testes from both surrogate species (KC ducks and WL males), used against KN purebred females, remained very close to the percent fertility realised from purebred KN chickens (controls). These initial results revealed from this trial study suggest definitively that, Kadaknath male gonads were readily accepted and grown inside the intra- and inter-species surrogate host, WL chicken and KC ducks, demonstrating a suitable intra- and inter-species donor-host system. Furthermore, the developed transplanted male gonads of KN chicken into the surrogates were found to have the potential to fertilise the egg and give rise to pure-line KN chicks.

## 1. Introduction

Unlike mammals, where semen and embryo cryo-freezing can serve the purpose of male and female germplasm conservation, respectively, in avian species, embryo cryopreservation is not feasible due to the morphology of the telolecithal avian eggs [1]. Similarly, semen conservation in birds has a variable success rate post-thaw due to the high sensitivity of the spermatozoa to the freezing-thaw cycle [2]. To overcome such challenges of in situ conservation of avian germplasm, ex-situ in vitro methods such as cryopreservation of early embryonic cells, blastodermal cells (BDCs), primordial germ cells (PGCs), pluripotent stem cells (PSCs) and induced pluripotent stem cells (iPSCs) [3] were developed and practised to preserve the target avian species germplasm. However, due to technical limitations in developing, establishing and standardising the PGC in vitro culture conditions for the enrichment and long-term culture of different avian species germ cells other than chicken, the application of PGCs in re-constituting a pure-line breed of chickens is still a challenge in the success of PGC mediated avian germplasm bio-banking [4].

Therefore, to overcome the above-mentioned technical challenges, transplantation of male and female gonadal tissues from a target avian donor species to a suitable recipient, whether intra- or inter-species, at an early developmental stage, is now gaining popularity since it was first demonstrated successfully by Song and Silversides [5,6,7]. Indian Kadaknath chicken, an indigenous breed, is highly pigmented and, therefore, becomes a model-of-choice organism for research related to genetics and developmental biology [8,9].

Earlier, we successfully demonstrated the harvest, in vitro culture and characterisation of circulating blood-derived PGCs from Kadaknath chicken [10,11]. Next, following the work conducted by Liptoi, et al., [12,13], we explored the potential of Kadaknath male gonads for transplantation in intra- and inter-species surrogate hosts, and further studied its potential to fertilise and regenerate the pure line KN chicks. This study is the first of its kind conducted in India and, therefore, helps in establishing a “proof-of-concept” for future work in the direction of conservation of gonadal tissue of other domestic, wild and endangered birds such as the Red Jungle fowl, the Great Indian bustard and the Vultures.

## 2. Materials and Methods

The Institutional Animal Ethics Committee (IAEC) of ICAR-DPR, RS, Bhubaneswar, Odisha (protocol code Dir/DPR/RS/02 dated 4 January 2021) approved all the experiments and interventions (methods).

### 2.1. Selection and Procurement of Avian Germplasm

White Leghorn (WL) and Kadaknath (KN), in form of one-day-old chicks, were procured from the Central Poultry Development Organization, Bhubaneswar, ODISHA, India. One-day-old ducklings of Khaki Campbell were procured from the ICAR-DPR, Regional Station, Bhubaneswar, ODISHA, India.

### 2.2. Sexing of Recipient Ducklings

One-day-old recipient chicks and ducklings were subjected to sex identification, employing manual vent sexing methods through trained personnel at the host laboratory.

### 2.3. Preparation of Donor Tissues

Male gonads were harvested from one-day-old Kadaknath chicks after euthanising them using cervical dislocation. The harvested gonads were kept in ice cold-Dulbecco’s Modified Eagle’s Medium (DMEM) for less than 2 h and transplanted onto the suitable host recipients.

### 2.4. Surgical Transfer of Gonadal Tissues

The recipient one-day-old WL chicks and KC ducklings were anesthetised using 0.5 mg of Ketamine and 0.1 mg of Xylazine per chick intramuscular injection. Feathers were removed using a razor blade, and a small incision was made to expose the peritoneal cavity, following procedures of Song and Silverside [6]. A keyhole size puncture was made through the rib cage, avoiding bleeding from an organ or tissue rupture. Small sections of 1–3 mm^3^ size of the donor gonads were transplanted without removing the yolk sac into the exposed cavity, grafting close to the endogenous gonads. The recipient’s gonads were left untouched. The intervened abdominal cavity was closed using Catgut sutures (size: 4–0).

### 2.5. Housing of the Birds

Immediately after surgical intervention, the ducklings and chicks were provided with oxygen supply, which helped them in faster recovery from anaesthesia effects. Antibiotic solution of 2.5 mg Monocef (injectable antibiotic 2.5 mg Ceftriaxone/chick, b.d.s, on day of surgery) followed by Cefixime (orally @ 0.2 mL b.d.s) was given to the recipient ducklings for 2 weeks. All the control and experimental birds’ post-surgical interventions were housed in plastic matted rearing cages for four weeks, respectively. The temperature, humidity and lighting for the housed birds were maintained according to their age. Water and feed were provided *ad libitum*, initially with the starter diet till the age of 16 weeks and thereafter with the pre-layer diet till 18 weeks and later on a layer diet. The cocks and the hens used for the artificial insemination experiments were housed separately starting from the 21st week onwards.

### 2.6. Use of Immunosuppressant

Mycophenolate mofetil was used as an immunosuppressant in a set of experiments, as described by Song and Silversides [5].

### 2.7. Removal of Transplanted Male KN Gonads from Chicken and Ducks

Post 14–20 weeks of transplantation, the gonads from the surrogate chicks and ducks were surgically removed, and seminal fluid was extracted to perform the artificial insemination.

### 2.8. Artificial Insemination Using the Fluid Extracted from Transplanted Gonads

Post harvesting of seminal fluid from the developed testicular tissues of KN, 15 randomly chosen KN hens (each) were identified, with 5 hens each subjected to AI, using 5 hens each per inseminated group using seminal fluids recovered from KC and WL surrogates each, besides usual AI employing one purebred KN male per 5 KN females as control.

### 2.9. Statistical Analysis

The experiments were organised following a completely randomised design that comprised 3 recipient groups of Kadaknath hens, which were subjected to experimental insemination. The resultant data (fertility and hatchability) were subjected to Analysis of Variance using SAS software package (SAS, Version 9.3).

## 3. Results

**Figure 1:**
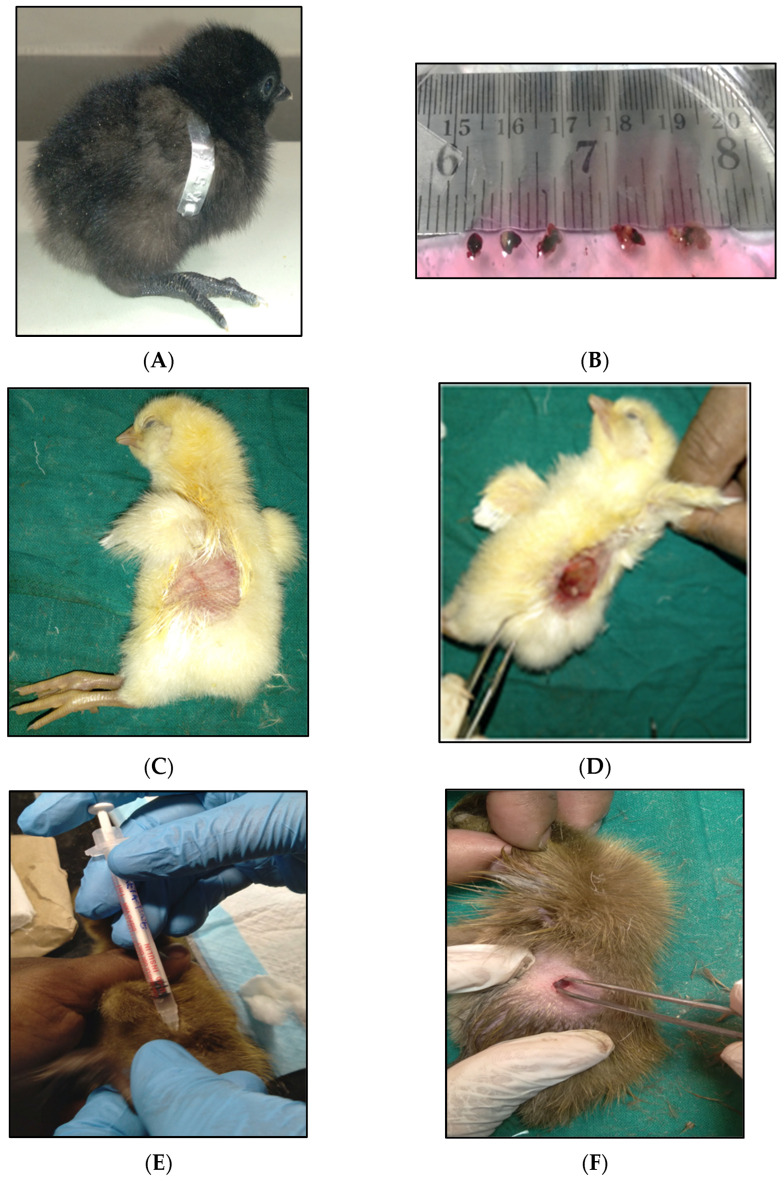

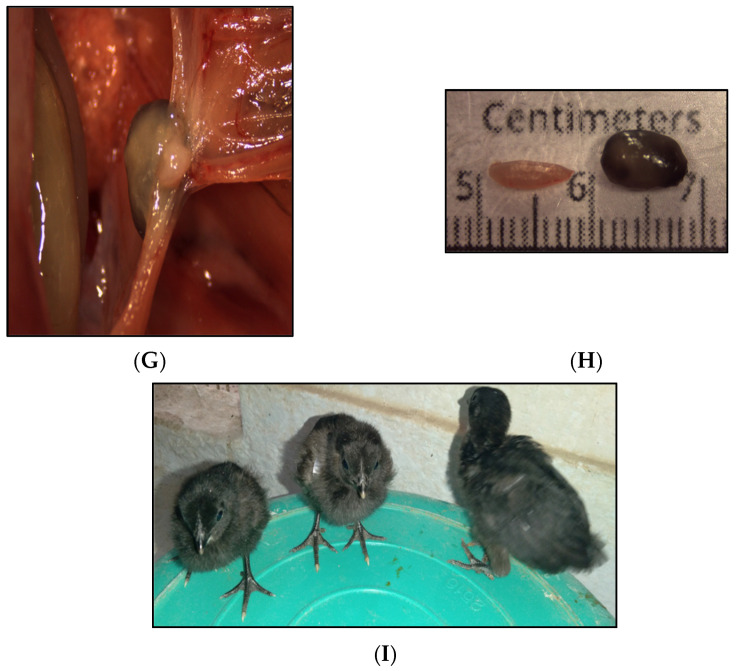
(**A**–**I**) shows the whole procedure of Kadaknath male gonad transplantation to chicken and duck as *intra- and inter-species* surrogate host systems serving bio-banking of poultry germplasm. (**A**) The male KN as donor. (**B**) The testes graft for transplantation. (**C**) One-day-old white leghorn chick as recipient. The feathers at the site of surgical intervention were removed using a razor. (**D**) Surgical transplantation of the testicular graft. (**E**) Administration of Ketamine and Xylazine as anaesthesia in KC as recipient, prior to surgery. (**F**) Surgical intervention in KC. (**G**) Development and vascular attachment of the transplanted KN testicular graft. (**H**) Size measurement of the developed testicular graft in white leghorn recipient compared to the endogenous testes. (**I**) Generation of pure-line KN chicks post-artificial insemination of the extracted fluid from the developed testicular implants.

Our initial results from the experiments suggest that Kadaknath male gonads can be successfully harvested from one-day-old chicks and further transplanted to one-day-old WL and KC ducklings. After 20 weeks post-operation, we found that the transplanted male Kadaknath testes (donor) were adhered to visceral parenchyma, in a near-random fashion, just above the endogenous chicken and duckling’s testes, respectively. The male Kadaknath testis (donor) was located close to the median plane, just above the kidneys. Figure 1A–H illustrates the whole procedure followed for transplantation of the KN testicular tissue grafts to WL and KC for bio-banking of valuable chicken germplasm. (A) show the one-day-old Kadaknath male chick as the donor model used in this study whose gonads were transplanted to surrogates. (B) The KN-harvested testes were kept in the ice-cold DMEM and cut into small grafts, which were used for the transplantation. (C) The recipient WL one-day-old chick was anesthetised, and feathers were removed from the site of surgical intervention. (D) The small KN testicular graft was surgically transplanted to the recipient WL chick through the punctured hole into the abdominal cavity. (E) Similarly, the recipient one-day-old KC duckling was anesthetised and prepared for the surgery. (F) After feathers were removed using a razor blade from the site of surgery in KC duckling, a keyhole puncture was made to surgically transplant the KN testicular graft (G) post-14 weeks of surgical transplantation of KN male gonadal tissue to the WL surrogate, the recipient chicken was sacrificed, and the vascular development and attachment of the transplanted Kadaknath testes was observed. (H) Morphology and size measurement of the surgically removed transplanted Kadaknath testes and endogenous testes was performed to study the development of transplanted tissue grafts. (I) The surgically removed KN donor testes from the WL and KC surrogates which developed significantly compared to the control were squeezed to collect the fluid [8] and used for artificial insemination experiments to pure-line female Kadaknath to produce pure-line Kadaknath chicks. Seminal extracts from each transplanted gonad were used for inseminating 5 KN females each, after which all resultant eggs were stored in an egg storage room before being incubated.

The results of fertility assessed from the hatching of the resultant eggs of each of the three categories, i.e., Controls [KN × KN] and the other 2 KN categories inseminated with seminal extracts from KC and WL surrogates, indicated nonsignificant differences in live chicks (reflected from Hatchability of 25.3% vs. 27.5%, on Total egg set basis, for the surrogate groups, respectively, as against the purebred KN showing up a figure of 40.9% only). When the details of unhatched eggs were analysed, including the embryos which ended up as dead-in-shells, from all the groups, it was clear that AI from such bio-banked semen entailed a fertility level of 62.5 vs. 68.7% for fertility from KN-origin transplanted testes, which did not vary significantly (*p* < 0.05) from the fertility of KN purebred eggs (72.5%). The summary of fertility and hatchability thus realised for the above sources of bio-banked semen and mentioned in Table 1.

## 4. Discussion

The initial results from the transplantation of male gonadal tissue grafts, i.e., testes, of Kadaknath one-day-old chicken to one-day-old WL chicken and one-day-old KC ducklings suggest that an intra- and inter-species bio-banking strategy can be successfully used to preserve the gonadal tissue responsible for germline transmission without removal of the yolk-sac in the recipient surrogate. Additionally, surgical removal of endogenous gonads may lead to excessive bleeding and can be a reason for the mortality of the recipient surrogate; the endogenous gonad can be left intact and does not interfere with the development of the donor testicular tissue. The anaesthesia worked efficiently, and we provided an oxygen supply to the operated recipients for their revival post-surgery. After initial mortality of up to 70% in the recipients post-surgery, we started providing a cocktail of antibiotics, which helped in maintaining 90–100% survivability.

The initial output of our experiments suggests that inter-species gonad transplantation is successful for a waterfowl to act as a surrogate host for such terrestrial domestic fowls of National significance, which entertains different environmental ecosystems. However, as per reports from other research groups, compatibility of a suitable donor and surrogate host avian species/breeds for orthotopic gonad transplantations remains a big challenge due to many undefined biological barriers.

The use of the seminal extract, squeezed out of transplanted testes in both WL and KC, as a reservoir of live spermatozoa of Kadaknath chickens, as verified from the fertility realised in AI of KN females, using seminal fluid from both surrogates, work out testimony that purpose of transplanting the said gonads, in intra- and inter- species recipients for bio-banking purpose. Despite the limitations of surgery-mediated gonad transplantation, which allowed a limited quantity of seminal yields, the proof of concepts in biobanking KN germplasm stands out as feasible and workable. Thus, this report supports the trends of earlier reports from lead scientists [8,13].

In conclusion, our studies demonstrated and established a “Proof-of-concept” that transplantation of chicken gonadal tissues (male component germplasm) is feasible and successful when dealt with a valued native chicken breed, Kadaknath, when performed surgically at neonatal stages of recipients, that can surpass the species barrier (across both intra and interspecies) as a whole. Further, it was evident that spermatogenesis also progressed with the simultaneous proliferation of the transplanted KN testes, in a physiologically convincing manner, with reasonable fidelity, needed for a successful bio-banking program. Such results call for further skill evolution and research in the surging gonad grafting arena so that the reproductive lives of functional gonads of valued chicken breeds can be enhanced indefinitely, with the possibility of extending such practices to other avian species.

## Figures and Tables

**Table 1:** The fertility and hatchability of the pureline chicks using bio-banked semen.

Source of Semen for Inseminating KN Hens	Total Eggs Set	% Fertility	% Hach (TES)	% Hach (FES)
Purebred KN Males	51	72.5	40.9	56.8
Khaki Campbell Surrogate Drakes	51	62.5	25.3	40.6
WL surrogate Cocks	51	68.7	27.5	40.0

N.B: TES and FES are abbreviations for hatchabilities realised on Total egg set basis (TES) and Fertile eggs basis (FES), respectively, which were generated from a uniform hatching program for all eggs. The fertility and hatchability, column-wise, did not differ significantly (*p* < 0.05) across germplasm sources.

## Data Availability

No new data were created or analyzed in this study. Data sharing is not applicable to this article.
